# Effect of SAMe-TT_2_R_2_ score and genetic polymorphism on the quality of anticoagulation control in Qatari patients treated with warfarin

**DOI:** 10.1007/s11239-020-02102-x

**Published:** 2020-04-10

**Authors:** Hazem Elewa, Iqrah Qurishi, Rawan Abouelhassan, Salam Abou Safrah, Eman Alhamoud, Loulia Bader

**Affiliations:** 1 Clinical Pharmacy and Practice Section, College of Pharmacy, QU Health, Qatar University, P.O. Box 2713, Doha, Qatar; 2 College of Pharmacy, QU Health, Qatar University, Doha, Qatar; 3 Alwakra Hospital, Doha, Qatar

**Keywords:** Same-TT_2_R_2_, Pharmacogenetics, Warfarin, Direct oral anticoagulants, Time in therapeutic range

## Abstract

There is no strong evidence on pharmacogenetics role on the quality of INR control after the initiation phase and on the maintenance of stable INR on the long term as measured by the time in therapeutic range (TTR). The benefit of a score such as SAMe-TT_2_R_2_ is that it can preemptively guide clinicians on whether to start the patient on warfarin or direct oral anticoagulant. To determine the association between genetic variants in *CYP2C9, VKORC1*, and *CYP4F2* and TTR. To validate SAMe-TT_2_R_2_ score predictive ability on the quality of anticoagulation in Qatari patients. This is an observational nested case–control study that was conducted on a cohort of Qatari patients treated with warfarin with previously identified genotype for the *CYP2C9, VKORC1,* and *CYP2F4*. The sample size of this cohort was 148 patients. Mean TTR was 62.7 ± 21%. TTR was not significantly different among carriers of the CYP2C9*2 &*3, VKORC1(–1639G>*A)* or CYP4F2*3 compared to their non-carriers alleles. None of the factors in the SAMe-TT_2_R_2_ score had a significant effect on the TTR except for the female gender where TTR was significantly lower in females (n = 89) compared to males (n = 59) (59.6 ± 21% vs. 67.2 ± 20%, p = 0.03). Furthermore, patients with SAMe-TT_2_R_2_ score of zero had significantly better TTR compared to those with higher scores (76.5 ± 17% vs. 61.8 ± 21%, p = 0.04). Logistic regression analysis showed that high SAMe-TT_2_R_2_ score was the only statistically significant predicting factor of poor INR control (odds ratio (OR) 5.7, 95% confidence interval (CI) 1.1–28.3, p = 0.034). Genetic variants have no contribution to the quality of INR control. SAMe-TT_2_R_2_ score was predictive for the poor quality of anticoagulation in a cohort of Qatari patients.

## Highlights

- SAMe-TT_2_R_2_ is a tool that can preemptively guide clinicians on whether to start the patient on warfarin or direct oral anticoagulant.
- There is no strong evidence on pharmacogenetics role on the quality of INR control and TTR after the initiation phase.
- This research shows that genetic variants have no contribution to the quality of INR control.
- Our results also indicate that SAMe-TT_2_R_2_ score is predictive for the poor quality of anticoagulation in a cohort of Qatari patients.

## Introduction

For over 60 years, warfarin has been the mainstay anticoagulant used in the prevention and treatment of thromboembolic complications in patients with atrial fibrillation, venous thromboembolism, prosthetic heart valves, and coronary artery disease [1]. Because of the narrow therapeutic index of warfarin and the substantial interpatient variability, careful monitoring of anticoagulation is necessary in order to minimize the risks associated with warfarin’s inadequate dosing and to ensure optimal outcomes for anticoagulated patients [2, 3]. Studies have shown that warfarin dose requirements, time to reach therapeutic level and risk of bleeding are influenced by demographic, environmental, clinical and genetic factors. In recent years, the substantial contribution of genetic variations has been well-defined [4, 5]. Specifically, several studies showed the considerable contribution of genetic variants in the genes encoding vitamin K epoxide reductase complex subunit-1 (VKORC1) and cytochrome p450 2C9 (CYP2C9) in warfarin dose variability. This contribution differs from one population to another depending on the allele frequencies for every population [6–9]. In recent work from our group on the effect of genetic polymorphisms on warfarin stable dose in Qatari patients, we have shown that *CYP2C9 and VKORC1* polymorphism accounted for 10.4% and 14.8% of warfarin dose variability, respectively [10].

There is compelling evidence on the usefulness of pharmacogenetics at the time of warfarin initiation in order to predict warfarin maintenance dose. However, to our knowledge, there is no evidence on the role of pharmacogenetics in the quality of INR control after the initiation phase and in the maintenance of stable INR on the long term as measured by the time in therapeutic range (TTR). TTR is an indicator of the quality of warfarin anticoagulation and is a surrogate marker for thromboembolism and bleeding clinical outcomes [11].

Since the approval of direct oral anticoagulants (DOACs) in 2010 [12, 13], their use has increased and they are now widely adopted by clinicians [13–18]. While DOACs were shown to have comparable efficacy and superior safety compared to warfarin in phase 3 trials [19–24], factors such as adherence and appropriate utilization may affect their perceived efficacy and safety. What makes these factors even more important in DOACs is the lack of a monitoring parameter and/or a surrogate marker to indicate their therapeutic level. Compared to warfarin, DOACs have more predictable therapeutic effect with a fixed-dose regimen, cause less intracranial bleeding, do not require routine monitoring, and have less drug–drug and drug–food interactions [19, 25, 26]. These benefits should also be considered in light of some potential disadvantages such as the increased risk of gastrointestinal side effects (especially for dabigatran and rivaroxaban), expense and lack of wide availability of antidote, and contraindication in patients with major renal dysfunction [19, 26–28]. Lastly, the cost of DOACs compared to warfarin may be prohibitive for many patients [29].

Among the efforts to aid clinicians in deciding whether to start or switch patients on warfarin versus DOACs is the use of SAMe-TT_2_R_2_ score. This score was derived to discriminate patients who would be less likely to achieve a good TTR with warfarin. In 2013, utilizing data of 2080 patients in the Atrial Fibrillation Follow-up Investigation of Rhythm Management (AFFIRM) trial, Apostolakis et al. developed the new SAMe-TT_2_R_2_ score (sex female, age <  60 years, medical history [more than two comorbidities], treatment [interacting drugs, e.g. amiodarone for rhythm control], tobacco use [doubled], race [doubled]) score). The score incorporates simple clinical and demographic factors that may influence anticoagulation control. It may also predict patients who may benefit from warfarin (achieving optimum anticoagulation control, as reflected by a good TTR above 65–70%; SAMe-TT_2_R_2_ score = 0–1) from those who may not (achieving low TTR and poor anticoagulation control; SAMe-TT_2_R_2_ score ≥ 2). It was further validated externally in a prospective cohort of patients receiving anticoagulant therapy, and it illustrated good discrimination performance in both the internal and external validation cohorts (c-index, 0.72; 95% CI 0.64–0.795; and c-index, 0.7; 95% CI 0.57–0.82, respectively) [30]. Thus, the benefit of a score such as SAMe-TT_2_R_2_ is that it can preemptively guide clinicians on whether to start the patient on warfarin or DOAC. In this study, we aim to validate the SAMe-TT_2_R_2_ score in a cohort of Qatari patients on chronic warfarin treatment and to determine the impact of genetic variants in *CYP2C9, VKORC1,* and *CYP2F4* on the level of INR control (by measuring TTR) at the maintenance phase (post first month of treatment). The ultimate objective is to determine if SAMe-TT_2_R_2_ score has good predictive ability of TTR in our cohort and if the incorporation of genetic polymorphism data can improve the predictive ability of the score.

## Methods

### Research design and ethics

This study is an observational nested case–control study that was conducted on a cohort of Qatari patients treated with warfarin at Hamad Medical Corporation (HMC) with previously identified genotype for the *CYP2C9*, *VKORC1*, and *CYP4F2*. Ethical approvals were obtained from the Institutional Review Board (IRB) of HMC, and from Qatar University (QU) IRB.

### Study setting and timeline

Patients were recruited from 3 different sites, all of which are part of HMC, the biggest medical institution in Qatar. These included the anticoagulation clinics at Al-Wakra Hospital, Heart Hospital, and Hamad General Hospital. Patients’ INR results were collected from the electronic health record (Cerner) for 1 year prior to patient enrollment in the genetic study which occurred between September, 2016 and March, 2017. For patients who were initiated on anticoagulation less than 1 year before their enrollment in the genetic study, INR results were only collected after the first month of warfarin treatment (to avoid the initiation phase) for 1 year afterwards or less depending on the duration of treatment.

### Study population and sampling

This study included warfarin-treated patients of Qatari nationality (identified as Qataris if they hold Qatari passport). Patients were considered eligible if they had been on warfarin for at least 6 weeks, had been on a stable warfarin dose for at least three consecutive clinic visits with their INR in therapeutic range, agreed to participate in the genetic study and future-related research and signed a written informed consent form. A stable warfarin dose was defined as a dose that did not vary by more than 10% between clinic visits [14]. Patients were excluded if they had liver cirrhosis, had advanced malignancies, were hospitalized within the previous 4 weeks or had a diarrheal or febrile disease within the previous 2 weeks of their enrollment in the genetic study. The sample size of this cohort was 148 patients.

### Data collection and outcome measures

In addition to the collection of INR readings as described above, baseline and clinical information including: age, gender, weight, smoking status, warfarin indication, concomitant medical conditions were collected. The primary outcome of our study was TTR which was calculated using linear interpolation method of Rosendaal et al. [11]. Genotyping data (previously collected) for (CYP2C9 *2 & *3) (rs1799853 and rs1057910, respectively), *VKORC1-1639G*>*A* (rs9934438) and for CYP4F2*3 (rs2108622) variants were presented. SAMe-TT_2_R_2_ score was calculated by providing one point for the following criteria: sex female, age < 60 years, medical history [more than two comorbidities], treatment [interacting drugs, e.g. amiodarone for rhythm control]. Two points were given to the following criteria: tobacco use and race (non-caucasians).

### Statistical analysis

Descriptive statistics was used to analyze baseline demographics. Depending on their normal distribution, numerical data were presented as mean with standard deviation or median and interquartile range. Categorical variables were presented as frequencies and percentages. For genetic variants, Chi-square-Goodness of Fit was used to make sure that all allele frequencies fit the Hardy–Weinberg equilibrium. Continuous variables were tested for normality tests including Kolmogorov–Smirnov and Shapiro–Wilk.

We used independent sample t-test to estimate the difference in mean TTR between the different genotype groups. SAMe-TT_2_R_2_ score (median, interquartile range) and TTR (mean, standard deviation) were calculated. The effect of SAMe-TT_2_R_2_ individual factors (example: gender, tobacco use etc.) as well as the effect of overall SAMe-TT_2_R_2_ (low vs. high) on TTR were assessed using independent sample t-test. Logistic regression was used to confirm the associated factors with poor quality of anticoagulation (performed twice using < 70% and < 65% as threshold for poor anticoagulation). Sensitivity, Specificity, positive predictive value and negative predictive value and odds ratio of SAMe-TT_2_R_2_ model on poor quality of anticoagulation were explored. A P value of less than 0.05 was considered statistically significant. All Statistical tests were carried using the IBM Statistical Package for Social Sciences, SPSS v. 26.0 (IBM Corp., Armonk, NY, USA).

## Results

### Study population characteristics

A total of 148 patients were included in the study. Patients’ mean age was 62.6 ± 13 years, 60% of them were female, and were mostly obese with an average body mass index (BMI) of 32 ± 6.9 kg/m^2^. Almost two third of the patients had atrial fibrillation as the main indication for warfarin, while diabetes mellitus and hypertension were the most common comorbidities in the cohort. Median (IQR) SAMe-TT_2_R_2_ was 2(1), while mean TTR was 62.7 ± 21%. Details of all demographics and baseline characteristics are shown in Table 1.

**Table 1:** Demographics and baseline characteristics

Variable	Total (N = 148)
Age (years) mean ± SD	62.6 ± 13
Gender no. (%)	
Female	89 (60.1)
BMI (kg m^−2^) mean ± SD	32 ± 6.9
Smoker no. (%)	11 (7.4)
Weekly warfarin dose (mg/week) median (IQR)	31.5 (21–43.7)
SAMe-TT2R2 score median (IQR)	2 (1)
SAMe-TT2R2 score (%)	
Zero	9 (6)
1	38 (25.5)
≥ 2	101 (68.5)
TTR mean ± SD	62.7 ± 21
TTR < 70% (%)	88 (59.5%)
Indication for warfarin no. (%)	
Atrial fibrillation	97 (65.5)
Valve replacement	23 (15.5)
Venous thromboembolism	19 (12.8)
Other*	9 (6.2)
Concomitant disease no. (%)	
Diabetes	84 (56.8)
Hypertension	98 (66.2)
HF	16 (10.8)
Cancer	3 (2)
Dyslipidemia	41 (27.5)
Concurrent medications no. (%)	
Statins	102 (68.9)
Antiplatelets	45 (30.4)
Antiarrythmics**	28 (18.9)
Thyroidal hormones	21 (14.2)
Genotype frequencies no. (%)	
*VKORC1*(-1639G>A)	
GG	39 (26.4)
AG	78 (52.7)
AA	31 (20.9)
CYP2C9*2 & *3	
*1*1	104 (70.3)
*1*2/*2*2	33 (22.3)
*1*3/*3*3	11 (7.4)
CYP4F2*3 (C > T)	
CC	50 (33.8)
CT	70 (47.3)
TT	28 (18.9)

*Thrombophilia, LV thrombus, or cardiomyopathy

**Amiodarone or digoxin

### Association of warfarin genetic variants with anticoagulation control (TTR)

There was no statistical significant difference in TTR between carriers and non-carriers of the minor allele in *CYP2C9*, *VKORC1* and *CYP4F2* (Table 2).

**Table 2:** Effect of pharmacogenetic variants on time in therapeutic range

PGX variant	TTR	P-value*
*CYP2C9* *2 & *3		
Carriers (n = 44)	66.9 ± 20.8%	0.108
Non-carriers (n = 104)	60.9 ± 21%	
*VKORC1* (-1639 G > A)		
Carriers (n = 107)	63.4 ± 21.2%	0.514
Non-carriers (n = 41)	60.8 ± 21.1%	
CYP4F2*3		
Carriers (n = 98)	64.5 ± 21.1%	0.145
Non-carriers (n = 50)	59.1 ± 20.9%	

*TTR* time in therapeutic range

*P value refer to the comparison of TTR between the carriers and the non-carriers of the genotype using independent sample T-test

### Association of SAMe-TT_2_R_2_ with anticoagulation control (TTR)

**Fig. 1:**
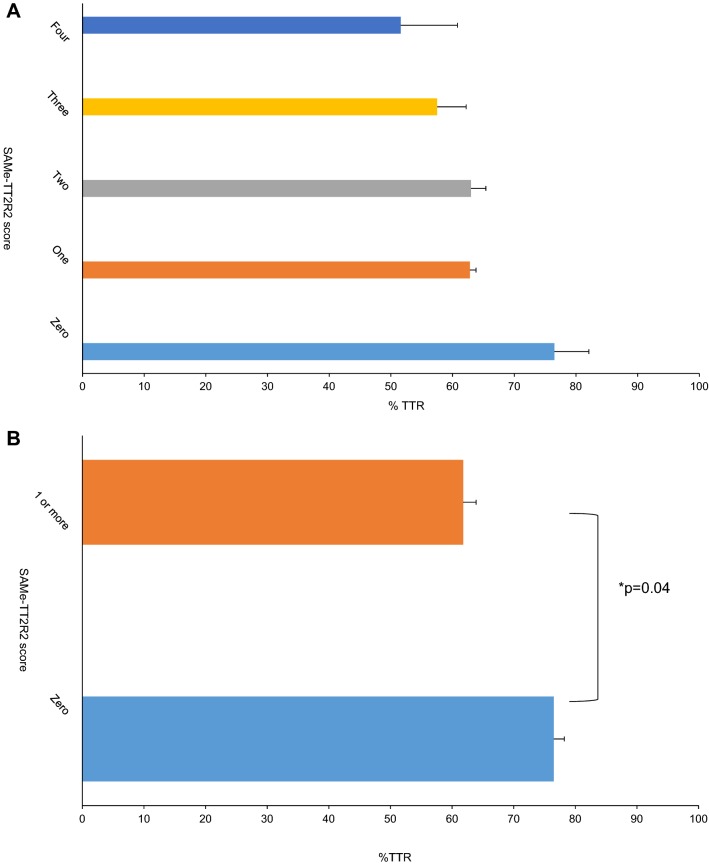
**a** Time in therapeutic range across different SAMe-TT_2_R_2_ scores. Bars represent time in therapeutic range (%TTR) and lines represent standard error of the mean across SAMe-TT_2_R_2_ scores. **b** Comparison between effect of high versus low SAMe-TT_2_R_2_ score on time in therapeutic range**.** Bars represent time in therapeutic range (%TTR) and lines represent standard error of the mean in patients with SAMe-TT_2_R_2_ score of zero versus a score of one or more. P value was measured using independent sample T-test

None of the factors in the SAMe-TT_2_R_2_ score had a significant effect on the TTR except for the female gender where TTR was significantly lower in females (n = 89) compared to males (n = 59) (59.6 ± 21% vs. 67.2 ± 20%, p = 0.03). Additionally, overall SAMe-TT_2_R_2_ score was associated with the level of anticoagulation control. However, results were only significant when a cut-off of one was used instead of two to indicate poor anticoagulation control (TTR was 76.5 ± 17% for patients SAMe-TT_2_R_2_ score of zero vs. 61.8 ± 21% for patients SAMe-TT_2_R_2_ score of one or more, p = 0.04) (Fig. 1a, b). Furthermore, a higher proportion of patients with TTR ≥ 65% and ≥ 70% were found in the low SAMe-TT_2_R_2_ score group compared to the high SAMe-TT_2_R_2_ score group (77.8% vs. 43.2%, p = 0.04; and 77.8% vs. 38.1%, p = 0.01). And to confirm that the overall SAMe-TT_2_R_2_ score is the predicting factor for the poor quality of anticoagulation (< 70%) rather than just the female gender, logistic regression analysis was performed and high SAMe-TT_2_R_2_ overall score was the only statistically significant predicting factor of the model (odds ratio (OR):5.7, 95% confidence interval (CI) 1.1–28.3, p = 0.034) (Table 3).

**Table 3:** Capability of SAMe-TT_2_R_2_ model to predict poor quality of anticoagulation across different cut-off points

	TTR < 70%	TTR < 65%
Sensitivity (%)	97.7	97.5
Specificity (%)	11.7	10.4
Positive predictive value (%)	61.9	56.8
Negative predictive value (%)	77.8	77.8
OR (95% CI)*	5.7 (1.1–28.3)	4.6 (0.92–23)
P value*	0.036	0.062

*OR* odd ratio, *CI* confidence interval

*OR (95% CI) and P value were measured using logistic regression

## Discussion

An important result from this study is that *CYP2C9, VKORC1,* and *CYP2F4* genetic mutations are not associated with the quality of anticoagulation during maintenance phase in a cohort of Qatari warfarin patients. Over the years, many have investigated the effect of genetic and non-genetic factors on warfarin dosing. Results have shown that the most important genes affecting warfarin dose among the different populations are the *CYP2C9, VKORC1* and *CYP4F2* [31]. Clinical utility trials were also conducted to investigate the ability of genetic-guided dosing to improve clinical outcomes during warfarin initiation and results were mostly positive [4, 5, 32]. On the other hand, there were very few studies that investigated the effect of these genetic factors on the long-term anticoagulation control with controversial results [33, 34]. In 2015, Park and colleagues collected data from 380 Korean patients with atrial fibrillation and evaluated genetic (*CYP2C9* and *VKORC1*) and non-genetic (SAMe-TT_2_R_2_ score) factors associated with TTR [33]. *VKORC1* 1173C>T was the only factor associated with TTR while there was no significant effect of SAMe-TT_2_R_2_ score on the quality of anticoagulation control. In 2018, another group from Spain investigated the effect of *VKORC1, CYP2C9∗2, CYP2C9∗3, MIR133A2* and SAMe-TT_2_R_2_ score on the level of anticoagulation control [34]. They tested 212 Spanish patients with nonvalvular atrial fibrillation treated with acenocoumarol and found that genetic factors did not have any significant effect on the quality of INR control. Beside SAMe-TT_2_R_2_, body mass index and regular vitamin K intake were the only predictors of poor anticoagulation control. Racial differences and the interaction effect with genetic mutations are among the factors that may have led to this contrast in the results between these two studies. Qataris and Arabs are considered Caucasians which may explain why results from our study were similar to the Spanish population rather than the Korean.

Additionally, results from our study have shown that gender was the only factor among the SAMe-TT_2_R_2_ criteria that was associated with the quality of anticoagulation in the same cohort. However, SAMe-TT_2_R_2_ overall score was associated with TTR and was the only predictor of poor quality of anticoagulation in the multivariate analysis but only when a score of one was used as a cut-off instead of two. Since SAMe-TT_2_R_2_ score was proposed and validated by Apostolakis and colleagues [30], external validation studies in populations from various countries took place and results were mostly positive [35–40]. Additionally, the score was tested in deep venous thrombosis as opposed to non-valvular atrial fibrillation patients and it was also shown to have a modest predictive ability for the quality of INR control [41, 42]. To the best of our knowledge, there were no previous studies looking at the validity of SAMe-TT_2_R_2_ score in Arabs. Our study which was performed on a cohort of Qatari patients taking warfarin for various indications is in line with the results from previous studies demonstrating the predictive ability of the SAMe-TT_2_R_2_ score and its promising clinical usefulness. While we were unable to demonstrate the association between the individual factors of the SAMe-TT_2_R_2_ score (apart from gender) and poor quality anticoagulation, the whole model still performed well when TTR of < 65% or < 70% were used as threshold for poor anticoagulation. We believe that this limitation is due primarily to the small sample size of the cohort used. Since one of the main objectives of this study was to determine the impact of genetic variants in *CYP2C9, VKORC1,* and *CYP2F4* on the level of INR control and the ability of these factors to improve the predictive ability of the SAMe-TT_2_R_2_ score, we restricted the inclusion in the study to Qatari patients with available genetic data.

Several limitations of the present study must be noted. The study had a small sample size. However, due to the limited number of Qatari patients on warfarin (about 1000 patients) [43], and the necessity to have genetic data for the included subjects, a larger sample was difficult to attain. As a retrospective study, control for bias and other potential confounding variables cannot be entirely eliminated. Lastly, we were unable to test the effect of SAMe-TT_2_R_2_ score on predicting efficacy and safety outcomes as this data was not reported consistently and was lacking accuracy.

In conclusion, SAMe-TT_2_R_2_ score was predictive for the poor quality of anticoagulation in a retrospective cohort of Qatari patients using warfarin. However, genetic factors were not associated with the quality of anticoagulation and did not add to the predictive ability of SAMe-TT_2_R_2_ score.
